# The impact of HIV infection on blood leukocyte responsiveness to bacterial stimulation in asymptomatic patients and patients with bloodstream infection

**DOI:** 10.7448/IAS.19.1.20759

**Published:** 2016-05-16

**Authors:** Michaëla A M Huson, Arie J Hoogendijk, Alex F de Vos, Martin P Grobusch, Tom van der Poll

**Affiliations:** 1 Center of Experimental and Molecular Medicine, Division of Infectious Diseases, Academic Medical Center, University of Amsterdam, Amsterdam, Netherlands; 2 Center of Tropical Medicine and Travel Medicine, Division of Infectious Diseases, Academic Medical Center, University of Amsterdam, Amsterdam, Netherlands; 3 Centre des Recherches Médicales de Lambaréné, Lambaréné, Gabon; 4 Institute of Tropical Medicine, University of Tübingen, Tübingen, Germany

**Keywords:** HIV, sepsis, cytokines, innate immunity, bacterial infections, leukocyte reprogramming

## Abstract

**Introduction:**

HIV-induced changes in cytokine responses to bacteria may influence susceptibility to bacterial infections and the consequent inflammatory response.

**Methods:**

We examined the impact of HIV on whole blood responsiveness to bacterial stimulation in asymptomatic subjects and patients with bacterial bloodstream infection (BSI). Whole blood was stimulated *ex vivo* with two bacterial Toll-like receptor agonists (lipopolysaccharide and lipoteichoic acid) and two pathogens (*Streptococcus pneumoniae* and non-typhoidal *Salmonella*), which are relevant in HIV-positive patients. Production of interferon-γ, tumour necrosis factor-α, interleukin-1β and interleukin-6 was used as a read-out.

**Results:**

In asymptomatic subjects, HIV infection was associated with reduced interferon-γ, release after stimulation and priming of the pro-inflammatory cytokine response to non-typhoidal *Salmonella*. In patients with BSI, we found no such priming effect, nor was there evidence for more profound sepsis-induced immunosuppression in BSI patients with HIV co-infection.

**Conclusions:**

These results suggest a complex effect of HIV on leukocyte responses to bacteria. However, in patients with sepsis, leukocyte responses were equally blunted in patients with and without HIV infection.

## Introduction

HIV patients have an increased risk of developing bacterial bloodstream infections (BSIs) and sepsis, which are associated with higher mortality [1–4]. Although the immunological mechanisms behind enhanced susceptibility, morbidity and mortality due to bacterial infections in HIV patients are incompletely understood, previous studies suggest a role for inadequate release of soluble mediators such as cytokines [5].

Pro-inflammatory cytokine release is an essential element of the host response during bacterial infection, which is important for protective immunity but also causes collateral damage due to exaggerated inflammation [6]. Sepsis is also associated with immune suppression, which involves a reduced ability of leukocytes to respond to re-stimulation with bacterial agonists [7,8]. Whole blood from sepsis patients without HIV co-infection demonstrated reduced capacity to release the pro-inflammatory cytokines tumour necrosis factor alpha (TNF)-α, interleukin (IL)-1β and IL-6 upon *ex vivo* stimulation, compared with blood from healthy subjects [9–11]. In contrast, previous investigations suggested that HIV infection results in priming of leukocytes to stimulation with bacterial agonists. *Ex vivo* stimulation of monocytes or peripheral blood mononuclear cells (PBMCs) from HIV patients with lipopolysaccharide (LPS) resulted in enhanced production of IL-1β, IL-6 and TNF-α [12,13].

Knowledge on the impact of HIV infection on the responsiveness of whole blood leukocytes to bacterial stimuli is limited, especially in the HIV endemic setting of sub-Saharan Africa. Furthermore, the effect of HIV on whole blood responsiveness during BSI is unknown. This information is relevant to understand host defences against bacteria in HIV patients and to obtain insight into the effect of HIV infection on hyper-inflammation and immune suppression during sepsis. Therefore, we examined (1) the whole blood leukocyte response in asymptomatic patients with HIV compared to healthy controls and (2) whether HIV co-infection influences sepsis-induced suppression of leukocyte responses to a secondary stimulus.

## Methods

### Patients

Blood cultures were obtained from adult patients (age ≥18 years) sequentially admitted to the Albert Schweitzer Hospital (Lambaréné, Gabon) between March 2012 and July 2013 with fever or hypothermia (body temperature ≥38°C or <36°C) and at least one other criterion of the systemic inflammatory response syndrome (tachycardia >90/min, respiratory rate >20/min or a white blood cell count <4×10^3^/mm^3^ or >12×10^3^/mm^3^). The cohort reported here was featured in part in previous studies on the impact of HIV infection on presentation and outcome of febrile illness, activation of the complement system and neutrophil extracellular traps [4,14,15]. Patients were included in the present analysis as soon as the blood culture became positive; at that moment, a second blood sample was drawn for whole blood stimulation as described below. Afebrile, asymptomatic controls, with or without HIV infection, were recruited in the vicinity of the hospital and the HIV outpatient clinic. This study was approved by the scientific review committee of the Centre des Recherches Médicales de Lambaréné. Written informed consent was obtained from all participants or their guardians.

### Clinical laboratory methods

Aerobic and anaerobic blood culture vials (Becton Dickinson, Franklin Lakes, NJ, USA) were incubated in the automated BD Bactec 9050 system (Becton Dickinson) for a maximum of five days or until the culture became positive. Standard culture-based methods were used for species identification (API test strips [bioMérieux, Craponnne, France] and BBL Enterotube or BBL Oxi/Ferm Tube [Becton Dickinson]). Coagulase-negative staphylococci and *Bacillus* spp. were routinely considered contaminants. *Streptococcus viridians* were regarded as contaminants as well, unless the patient had clinical signs of endocarditis or meningitis. As part of a clinical trial requirement, the microbiology laboratory at the Albert Schweitzer Hospital successfully participates in regular external quality assurance programmes addressing species identification. For HIV testing, a rapid test was used (Vikia HIV 1/2 (bioMérieux) or Determine^TM^ HIV 1/2 (Alere, Yavne, Israel), depending on local availability). In case of a positive reading, the result was confirmed by VIDAS HIV DUO Ultra (bioMérieux) and ImmunoComb HIV 1&2 BiSpot (Alere). Viral loads were measured in EDTA plasma using Cobas Amplicor HIV-1 Monitor Test, v1.5 (Roche, Pleasanton, CA, USA). The detection limit of this assay was 200 copies/ml. CD4 counts were determined using BD FACSCount (Becton Dickinson). Creatinine, aspartate transaminase (ASAT) and alanine transaminase (ALAT) were determined by the hospital laboratory in all patients and controls. Glomerular filtration rates were estimated by the modification of diet in renal disease (MDRD) formula [16]. Renal failure was defined by a glomerular filtration rate <60 ml/min/1.73 m^2^, and liver injury was defined by both ASAT and ALAT greater than two times the upper limit.

### Whole blood stimulation and assays

Stimulations in BSI patients and controls were done on one occasion. Asymptomatic subjects were sampled on inclusion into the study. In patients with BSI, material for whole blood stimulation was obtained immediately after the blood culture became positive, mostly (in 88% of cases) within one day after admission. Heparinized whole blood was mixed with an equal volume of Roswell Park Memorial Institute (RPMI) 1640 medium (Sigma-Aldrich, Saint Louis, MO, USA) with or without LPS (*Escherichia coli* 0111:B4, 100 ng/ml, InvivoGen, San Diego, CA, USA), lipoteichoic acid (LTA) (*Staphylococcus aureus*, 10 µg/ml, InvivoGen), heat-killed *Streptococcus pneumoniae* serotype 3 (ATCC6303) or heat-killed *Salmonella enterica* serovar Typhimurium, strain 14028 (NTS, non-typhoid *Salmonella*; end concentrations equivalent to 10^3^ colony forming units/Litre or 100 colony forming units/Litre, respectively), and incubated for 24 hours in a 5% CO_2_ incubator at 37°C, after which supernatants were harvested and stored at −80°C. To establish the optimal dose of heat-killed bacteria, LPS and LTA, to induce a robust cytokine response, we performed whole blood stimulations with serial dilutions of stimuli, using blood from healthy volunteers. Interferon (IFN)-γ, TNF-α, IL-1β and IL-6 were measured by cytometric bead array (BD Biosciences, San Jose, CA, USA). Each cytokine assay was done for all samples in one run on the same day using the same batch of reagents, thereby eliminating inter-assay variability.

### Statistical analysis

Categorical variables are presented as percentages and continuous variables as medians with their interquartile range in the table and as box-and-whisker plots in the figures. We used Fisher's exact tests for comparisons of categorical variables, Mann–Whitney U tests or Kruskall–Wallis tests to assess differences for non-normally distributed continuous variables, and unpaired *t*-tests or one-way ANOVA tests for normally distributed variables. Cytokine release in response to a stimulus was determined by calculating the difference in cytokine levels between the stimulated and medium control samples. When this resulted in a negative value, samples were considered tolerant to stimulation and were given a default value of 1 pg/ml. Outliers were determined using a Grubbs test. Samples with outliers in the unstimulated control were excluded from our analyses. An additional sensitivity analysis was performed in some cases to correct for differences in leukocyte count by dividing cytokine read-outs by the number of leukocytes. Data analyses and creation of figures were done with GraphPad Prism (GraphPad Software, La Jolla, CA, USA). A *p*-value of <0.05 was applied as the level of significance in all analyses. This was an exploratory study, so a formal sample size calculation could not be performed. No mathematical correction was made for multiple comparisons.

## Results

### Patient characteristics

Table 1 presents the baseline characteristics of the study population. We recruited 60 asymptomatic HIV patients and 35 HIV-negative controls. In addition, we obtained blood cultures from 466 patients, 33 of which revealed a bacterial pathogen, including 14 patients with HIV infection.

**Table 1:** Baseline characteristics of the study populations

	Asymptomatic subjects					Bloodstream infection			
		
	Total *n*=95	HIV− *n=*35	HIV+ on cART *n=*34	HIV+ untreated *n=*26	*p*^a^	Total *n=*33	HIV− *n=*19	HIV+ *n=*14^b^	*p*^a^
Demographics									
Age (years)	40 (31 to 50)	38 (28 to 43)	46 (36 to 54)	38 (32 to 49)	0.85	37 (26 to 47)	28 (23 to 56)	39 (32 to 47)	0.37
Male sex, *n* (%)	37 (38.9)	17 (48.6)	12 (35.5)	8 (30.8)	0.32	11 (33.3)	6 (31.6)	5 (35.7)	1.0
Laboratory parameters									
Leukocyte count (10^9^/L)	4.7 (3.4 to 5.5)	5.1 (4.5 to 6.3)	4.0 (3.0 to 4.8)	4.4 (3.1 to 5.4)	**0.001**	8.6 (5.0 to 7.5)	12.4 (4.6 to 22.5)	8.0 (5.1 to 15.0)	0.44
Renal failure, *n* (%)^c^	1 (1.1)	1 (2.9)	0 (0)	0 (0)	NA^d^	13 (46.4)	6 (40.0)	7 (53.8)	0.71
Liver injury, *n* (%)^c^	0 (0)	0 (0)	0 (0)	0 (0)	NA^d^	5 (17.9)	3 (20.0)	2 (15.4)	1.0
CD4 counts (cells/mm^3^)	–	–	302 (189 to 480)	461 (320 to 642)	**0.03**	–	–	150 (47 to 308)	–
HIV load (copies/ml)^e^	–	–	200 (200 to 200)	0.13e6 (200 to 0.69e6)	**0.0001**	–	–	8.11e6 (1.76e6 to 30.00e6)	–
Antimicrobial treatment at time of sampling									
Antibiotic treatment	–	–	–	–	–	26 (78.8)	12 (63.1)	14 (100)	0.01
Antimalarial treatment	–	–	–	–	–	8 (24.2)	7 (36.8)	1 (7.1)	0.10

Data are presented as medians (interquartile ranges), except for sex, renal failure, liver injury and antibiotic treatment; *p*-values below 0.05 are depicted in bold

a *p*-values indicate statistical significance within groups (asymptomatic or with BSI) between different categories

b this group included three patients on cART (not stratified because of the low sample size)

c data on renal and liver injury were missing for one HIV+ patient and four HIV− patients. Percentages were calculated using the total number of patients for whom data was available.

d Statistical comparison was not possible

e HIV loads below the detection limit (200 copies/ml) were set at 200 copies/ml; BSI, bloodstream infection; cART, combination antiretroviral therapy; NA, not applicable.

Age and sex distribution did not differ according to HIV status in either asymptomatic subjects or patients with BSI. There were no significant differences in renal or liver injury according to HIV status, but patients with a BSI and HIV co-infection were more likely to be on antibiotics at the time of sampling compared to HIV-negative BSI patients (Table 1). The most common antibiotics used were amoxicillin/clavulanate (*n*=10), ceftriaxone (*n*=7) and ciprofloxacin (*n*=5). Of asymptomatic HIV patients, 57% (*n*=34) were on combination antiretroviral therapy (cART), consisting of two nucleoside reverse transcriptase inhibitors combined with either a non-nucleoside reverse transcriptase inhibitor (80%) or a protease inhibitor (20%); the majority of these patients had undetectable HIV loads in their blood (<200 copies/ml). HIV patients with a BSI had higher HIV loads and lower CD4 counts when compared with asymptomatic HIV patients (both *p*<0.01). In this group, only three (21%) patients were on cART. The abdomen (*n*=8) and lungs (*n*=7) were the most common sites of infection in patients with a positive blood culture, with no significant differences according to HIV status. The causative pathogens are depicted in Table 2. There were no differences in pathogens according to HIV status, except for infection with *S. pneumoniae*, which was found exclusively in HIV-positive patients.

**Table 2:** Sites of infection and causative pathogens in patients with bloodstream infection

	Total *n*=33	HIV− *n*=19	HIV+ *n*=14	*p*
Site of infection (%)				
Abdominal infection	8 (24.2)	6 (31.6)	2 (14.3)	0.42
Pneumonia	7 (21.2)	2 (10.5)	5 (35.7)	0.11
Skin or soft tissue infection	2 (6.1)	0 (0)	2 (14.3)	0.17
Urinary tract infection	6 (18.2)	3 (15.8)	3 (21.4)	1.0
Primary bacteraemia	10 (30.3)	9 (47.4)	1 (7.1)	**0.02**
Meningitis	1 (3.0)	0 (0)	1 (7.1)	0.42
Pathogens (%)				
*Escherichia coli*	10 (30.3)	7 (36.8)	3 (21.4)	0.46
*Staphylococcus aureus*	4 (12.1)	1 (5.3)	3 (21.4)	0.29
*Streptococcus pneumoniae*	4 (12.1)	0 (0)	4 (28.6)	**0.02**
*Salmonella typhi*	2 (6.1)	2 (10.5)	0 (0)	0.50
Non-typhoidal *Salmonella*	2 (6.1)	0 (0)	2 (14.3)	0.17
*Klebsiella pneumoniae*	1 (3.0)	1 (5.3)	0 (0.0)	1.0
*Serratia marcescens*	1 (3.0)	1 (5.3)	0 (0.0)	1.0
*Streptococcus viridans*	1 (3.0)	0 (0)	1 (7.1)	0.42
B-hemolytic streptococci^a^	8 (24.2)	7 (36.8)	1 (7.1)	0.10

*P*-values below 0.05 are depicted in bold

a Including group B (3), group C (3) and group D (2) streptococci. The single HIV-positive patient in this group was infected with a group C streptococci.

### Whole blood stimulations

**Figure 1:**
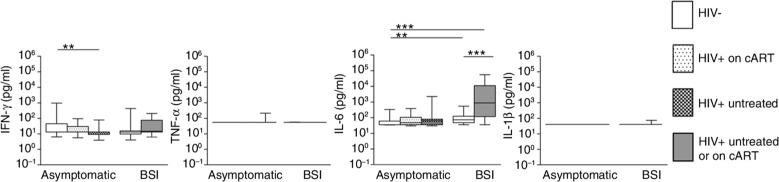
Spontaneous cytokine release by whole blood samples from BSI patients and asymptomatic subjects with or without HIV infection. Whole blood was kept in RPMI medium for 24 hours. Data are depicted as box-and-whisker plots depicting the smallest observation, lower quartile, median, upper quartile and largest observation. Significant differences between HIV-negative asymptomatic controls and asymptomatic HIV patients or sepsis cases are shown, as well as differences within groups (asymptomatic or sepsis) according to HIV status. The group of HIV-positive patients with BSI included three patients on cART (not stratified because of the low sample size). For TNF-α and IL-1β, almost all samples were below the limit of detection. These samples are depicted at the limit of detection (55 and 40 pg/ml, respectively). *p<0.05, *p<0.01, ***p<0.001. BSI, bloodstream infection; cART, combination antiretroviral therapy; TNF-α, tumour necrosis factor alpha; IL-1β, interleukin-1β.

We performed whole blood stimulations with two bacterial Toll-like receptor (TLR) agonists (LPS, a TLR4 agonist, and LTA, a TLR2 agonist [17]) and two pathogens (*S. pneumoniae* and NTS) relevant for HIV-positive patients [1]. Two asymptomatic HIV patients had to be excluded from analysis due to outliers in the unstimulated control samples. Cytokine releases from unstimulated whole blood (medium control samples) are depicted in Figure 1.

**Figure 2:**
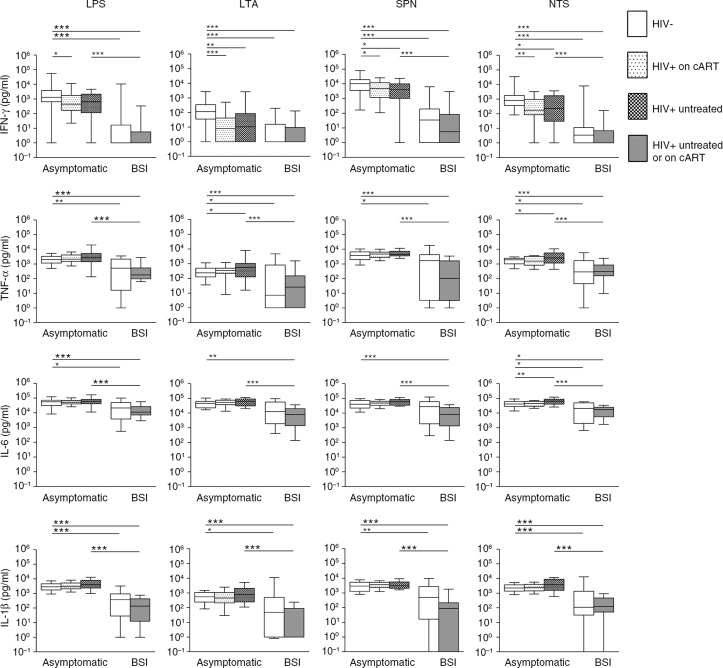
Cytokine release by whole blood stimulated with bacterial cell wall components (LPS or LTA) or bacteria (*S. pneumoniae* or NTS). Whole blood was stimulated for 24 hours. Data are depicted as box-and-whisker plots depicting the smallest observation, lower quartile, median, upper quartile and largest observation. Cytokine release in response to a stimulus was determined by calculating the difference in cytokine levels between the stimulated and medium control samples. Samples without additional cytokine release in response to stimulation compared to medium control were set at 1 pg/ml to allow for presentation on a logarithmic scale. Significant differences between HIV-negative asymptomatic controls and asymptomatic HIV patients or sepsis cases are shown, as well as differences within groups (asymptomatic or sepsis) according to HIV status. The group of HIV-positive patients with BSI included three patients on cART (not stratified because of the low sample size). *p<0.05, **p<0.01, ***p<0.001; BSI, bloodstream infection; cART, combination antiretroviral therapy; LPS, lipopolysaccharide; LTA, lipoteichoic acid; SPN, *Streptococcus pneumoniae*; NTS, non-typhoidal *Salmonella*.

Upon incubation with all tested bacterial stimuli, blood obtained from asymptomatic HIV patients produced less IFN-γ when compared with blood from HIV-negative healthy controls, irrespective of cART use (Figure 2). To examine whether this finding was related to lower leukocyte counts in asymptomatic HIV patients, a sensitivity analysis was performed in which results were corrected for leukocyte counts. The effect of HIV remained significant for LTA and NTS, suggesting a true effect of HIV on whole blood leukocyte IFN-γ release. In response to NTS, blood from asymptomatic HIV patients not treated with cART released higher amounts of the pro-inflammatory cytokines TNF-α and IL-6, with a similar trend for IL-1β (*p=*0.08). Incubation with other stimuli did not result in enhanced pro-inflammatory cytokine release by blood leukocytes from HIV patients, except for TNF-α secretion in response to LTA (Figure 2).

As expected [8–11], in patients with BSI, LPS-induced release of IFN-γ, TNF-α, IL-1β and IL-6 was reduced compared to asymptomatic subjects. Likewise, cytokine induction by NTS was attenuated in blood from BSI patients. This immunosuppressive effect of BSI was also observed in blood stimulated with the gram-positive stimuli LTA and *S. pneumoniae*, but was less consistent for IL-6 release. Similar results were observed when comparing HIV-positive BSI patients with asymptomatic HIV-positive patients (Figure 2). HIV infection did not influence the cytokine production capacity of blood leukocytes in the presence of BSI (Figure 2).

## Discussion

The capacity of leukocytes to respond to bacterial agonists is a strong denominator of both the early protective immune response and the late, potentially damaging inflammatory reaction. In addition, the reduced ability of leukocytes to react to bacterial agonists during established severe infection has been implicated as an important feature of immune suppression in patients with sepsis [7]. We hypothesized that HIV infection might influence the cytokine production capacity of blood leukocytes and studied this in asymptomatic subjects and patients with BSI. Our main findings are that, in asymptomatic subjects, HIV infection was associated with reduced IFN-γ release in response to bacterial stimulation, irrespective of cART use, whereas there was priming of the pro-inflammatory cytokine response to NTS. HIV infection had no influence on the diminished cytokine production capacity of blood leukocytes from BSI patients.

The reduced IFN-γ response to stimulation observed in asymptomatic HIV patients could be related to their white blood cell count, which was significantly lower than in HIV-negative controls. However, after correction for leukocyte counts the effect of HIV remained significant for LTA and NTS. Furthermore, this immunosuppressive effect was not observed for other cytokines, suggesting an IFN-γ-specific mechanism. NK cells, the main producers of IFN-γ, were previously shown to be less responsive to *ex vivo* stimulation with *E. coli* or NTS in HIV-positive patients [18]. Furthermore, IFN-γ production in response to pneumococcal antigens was reduced in CD4 T cells from HIV patients [19]. Our findings extend the relevance of these findings to a whole blood stimulation model and a wider range of bacterial agonists. Several mechanisms could be involved in reduced NK cell responsiveness in HIV patients, including the expansion of an unresponsive subset of NK cells and shedding of MHC class I chain-related molecules, which provide negative feedback to NK cells [5]. In addition, reduced numbers and impaired function of CD4 and CD8 T cells, which also produce IFN-γ [20], could play a role in the impaired IFN-γ response. As IFN-γ deficiency is associated with enhanced susceptibility to intracellular bacterial infections [21], an attenuated IFN-γ response to stimulation may contribute to the enhanced susceptibility of HIV patients to infections with NTS.

We observed no significant differences in whole blood leukocyte responses between asymptomatic patients with and without cART. A possible explanation for the absence of improvement on cART is the presence of more advanced disease in patients on cART, as illustrated by their lower CD4 counts (Table 1).

We found no evidence for a primed response to LPS in the whole blood of asymptomatic HIV patients, as previously described after stimulation of monocytes and PBMCs [12]. However, we did observe consistent priming of pro-inflammatory cytokine release after stimulation with NTS in asymptomatic HIV patients without cART as compared to healthy controls. In line with this finding, a previous study found that HIV infection was associated with enhanced cytokine release from alveolar macrophages in response to NTS [22]. Furthermore, analysis of gene expression profiles of HIV patients with NTS BSIs showed a lack of coordinated inflammatory response, which was not observed for other bacterial pathogens, suggesting a unique interaction between NTS and HIV [23]. The enhanced susceptibility of HIV patients to invasive NTS suggests that enhanced pro-inflammatory cytokine release upon NTS exposure is not protective, but may contribute to more extensive tissue damage.

In patients with BSI, HIV co-infection had no impact on the capacity of blood leukocytes to release cytokines, regardless of the cytokine read-out or stimulus applied. In line with this, we observed a predominantly common genomic response of whole blood leukocytes in Dutch ICU patients with or without sepsis [24]. These results suggest a predominantly common host response in sepsis patients with or without HIV co-infection.

Our study was limited by the relatively small number of patients with BSI, so we grouped patients with different pathogens and sites of infection. Possibly as a consequence, variance in leukocyte responses within groups was relatively large. Differences in antibiotic treatment regimens was another potential source of variance. In order to avoid a type I error, we did not correct for multiple comparisons. However, by testing different bacterial stimuli and different read-outs, we were able to observe consistency in leukocyte responses in different patient groups.

## Conclusion

To the best of our knowledge, this is the first study to examine the impact of HIV on leukocyte responsiveness in patients with BSI. In line with previous studies, we found that responses to NTS, a very relevant pathogen in the context of HIV infection, were most affected by HIV. Our results exemplify the complex interactions between HIV and bacteria that enter the bloodstream in an era where “common” pathogens now dominate the spectrum of causative organisms in BSIs in HIV-positive patients.
